# Improvement of pain related self management for oncologic patients through a trans institutional modular nursing intervention: protocol of a cluster randomized multicenter trial

**DOI:** 10.1186/1745-6215-11-29

**Published:** 2010-03-22

**Authors:** Patrick Jahn, Maria Kitzmantel, Petra Renz, Ene Kukk, Oliver Kuss, Anette Thoke-Colberg, Ingrid Horn, Margarete Landenberger

**Affiliations:** 1 Institute for Health and Nursing Science, Medical Faculty, Martin-Luther-University Halle-Wittenberg, Germany; 2 University Hospital rechts der Isar, Technical University Munich, Germany; 3 Institute for Medical Epidemiology, Biostatistics, and Informatics, Medical Faculty, Martin-Luther-University Halle-Wittenberg, Germany; 4 University Hospital Halle, Martin-Luther-University Halle-Wittenberg, Germany

## Abstract

**Background:**

Pain is one of the most frequent and distressing symptoms in cancer patients. For the majority of the patients, sufficient pain relief can be obtained if adequate treatment is provided. However, pain remains often undertreated due to institutional, health care professional and patient related barriers. Patients self management skills are affected by the patients' knowledge, activities and attitude to pain management. This trial protocol is aimed to test the SCION-PAIN program, a multi modular structured intervention to improve self management in cancer patients with pain.

**Methods:**

240 patients with diagnosed malignancy and pain > 3 days and average pain ≥ 3/10 will participate in a cluster randomized trial on 18 wards in 2 German university hospitals. Patients from the intervention wards will receive, additionally to standard pain treatment, the SCION-PAIN program consisting of 3 modules: pharmacologic pain management, nonpharmacologic pain management and discharge management. The intervention will be conducted by specially trained oncology nurses and includes components of patient education, skills training and counseling to improve self care regarding pain management beginning with admission followed by booster session every 3rd day and one follow up telephone counseling within 2 to 3 days after discharge. Patients in the control group will receive standard care.

Primary endpoint is the group difference in patient related barriers to management of cancer pain (BQII), 7 days after discharge. Secondary endpoints are: pain intensity & interference, adherence, coping and HRQoL.

**Discussion:**

The study will determine if the acquired self management skills of the patients continue to be used after discharge from hospital. It is hypothesized that patients who receive the multi modular structured intervention will have less patient related barriers and a better self management of cancer pain.

**Trial Registration:**

ClinicalTrials NCT00779597

## Background

Pain is still one of the most frequent and distressing symptoms in cancer patients particularly in advanced stages of disease [1-3]. A currently published survey on symptom prevalence in more than 1300 cancer patients with various malignancies showed that almost 85% suffer from pain [4]. Pain was not only a frequent symptom but had also high intensity with a mean score of 5.0 (range 2.0 - 7.0 on a 0 to 10 NRS). Untreated and persistent pain interferes with patients' activities of daily living and reduces health related quality of life (HRQoL) [5].

For up to 90% of patients, sufficient pain relief can be obtained if adequate guideline based treatment is provided [6]. However, pain often remains undertreated due to patient related, institutional or health care professional barriers [7]. Therefore sufficient treatment of pain and associated symptoms must be based on receptive attitudes and effective self management of the patient, facilitated through patient education and counseling.

Patient related barriers include attitudes and believes about pain treatment in four dimensions: firstly misconceptions of physical effects of pain medication, such as that medication cannot really control pain, medication should be saved in case the pain gets worse or side effects of pain medication are difficult to control; secondly fatalism, e.g. the concerns that the pain treatment is ineffective; thirdly communication related barriers, e.g. not annoying the professionals by complaining about pain or distracting the physician from treating the disease by paying attention on pain treatment; fourthly worries about harmful effects of pain medication, e.g. that pain medication is very addictive. These attitudes and believes are held by almost all patients and build barriers for adequate pain control [13,16].

The evidence on psycho educational interventions to reduce patient related barriers is limited. Four RCTs have tested the effectiveness of such programs [8-11]. None of these trials implemented the intervention in an inpatient setting and two suffer from small sample size [8,11]. Furthermore, generalisability of these results to clinical settings is limited, due to a low degree of involvement of regular ward staff in application of study intervention, and application only in cancer subtypes (e.g. gynecological cancers).

Therefore the aim of this study is the evaluation of Self Care Improvement through Oncology Nursing, SCION-PAIN a multi modular nursing administered program. The program includes interventions to optimize pharmacological and nonpharmacological pain treatment, additionally to pain related discharge management and counseling to reduce patients' barriers and improve pain management. We will compare the effectiveness of the intervention on a trans institutional path, starting with an inpatient setting and continuing to home setting after discharge.

To avoid contamination, the intervention will be applied at the level of wards and the study is designed as a cluster randomized clinical trial.

## Methods

In two German university hospitals, eighteen inpatient wards with each at least 10% of patients diagnosed with cancer will be randomized. Patients will be included if they are aged 18 to 80 years, have a cancer diagnosis, an average pain intensity score of ≥ 3.0 (on a 0 to 10 NRS), pain persistence for more than 3 days, the ability to read, write, and understand German, a scheduled follow up appointment at the clinic, and signed written informed consent.

Patients will be excluded if they have a limited performance status (ECOG 4), documented ongoing alcohol or drug abuse, surgery within the last 3 days or showing disorientation regarding date, place and situation.

The study will be performed according to the ICH-GCP principles and was approved by the regional ethics committees of Martin-Luther-University Halle-Wittenberg and Technical University Munich.

### Interventions

The SCION-PAIN program is aimed to reduce patient related barriers to pain management in order to decrease pain intensity and pain interference with daily activities, as well as improving adherence and HRQoL. The program was developed by a part of the authors based on extensive literature review on efficient pain management. SCION-PAIN program includes three modular algorithm based protocols summarized in a handbook for the professionals, supplemented by a teaching booklet tailored to the patients' needs, a patient held record, a discharge self preparation checklist, and a compact disc with PMR exercise [12].

The intervention is structured into three modules. The first module "Pharmacologic pain management" addresses adequate pain assessment, effective communication about pain and administration of pain medication. The second module "Nonpharmacologic pain management" includes information about the effectiveness of complementary pain treatment methods and the application of a compact disc based PMR exercise. The third module "Pain related discharge management" provides advice on how to maintain the self management strategies learned in module one and two after discharge. Additionally, a checklist to ensure adequate discharge management is administered. This checklist contains seven essential questions to be answered during hospitalization, e.g. who will prescribe me further pain medication at home or who will provide advice in case of inappropriate pain management. The checklist will be assessed for completeness by the research nurse one day before discharge. The third module is aimed to prepare patients to detect possible situations of inappropriate self management and to counteract appropriately.

The content of all three modules is summarized for the patient within the 33-paged education booklet "Leave the pain behind you". Each pain related topic is specially enriched with information regarding patient related barriers to pain management, according to the domains identified by Ward [11] and Gunnarsdottir [13]. For example, a frequent patient related barrier is the reluctance to report pain in order to be a "good patient". Addressing this problem, patients get specific information about the importance of communicating pain in order to provide the best possible pain management. The booklet was developed by a part of the authors based on a literature review and approved by an institutional advisory panel of pain experts.

The administration of the modules will be individually planned for each patient in the intervention group depending on her or his educational needs. These demands will be assessed through a process documentation to ensure a homogenous intervention. The documentation will allocate the patients into one of three groups (low, intermediate and high resources for self management) consistent with their resources regarding knowledge, self care actions, and attitudes. Accordingly, each module can be fully, partially or not at all applied on the patient. The assessment of educational needs will be revised before and after every follow up counseling throughout the course of the intervention.

**Figure 1:**
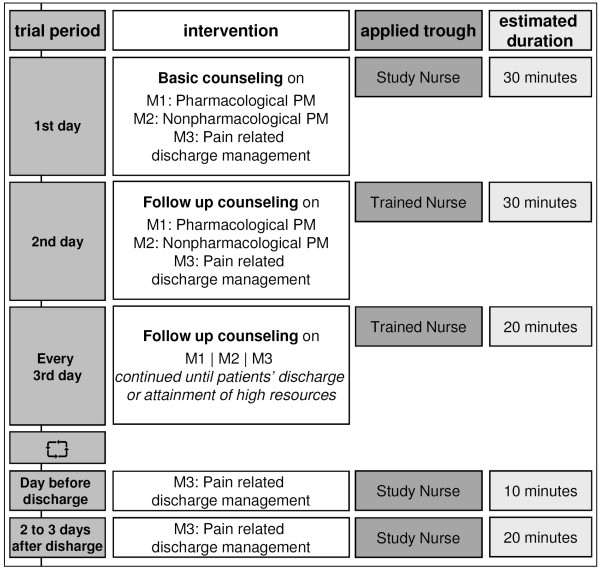
**SCION-PAIN program intervention structure**.

The first session will be held by the research nurse within 24 h after trial inclusion containing an overall introduction on self management of pain based on the booklet handed out to each patient (see figure 1).

Continuing the educational program the day after first education took place, the follow up sessions is going to be carried out by specially trained ward nurses. Unless the patient calls for further educational needs, the following counseling sessions will be scheduled every third day until patients' discharge or attainment of high resources for self management regarding knowledge, self care actions, and attitudes.

The intervention will end with a final obligatory application of a part of module three comprising a follow up telephone counseling carried out by the research nurse within 2 to 3 days after discharge. The counseling is based on a telephone guideline [14] and addresses additional issues to the previous session, as indicated in the process documentation.

Prior to the implementation, all modules were critically approved by an expert panel including nurses of the participating hospitals and scientific experts.

In the intervention group, the program will be administered by specially trained ward nurses in cooperation with a research nurse. Nurses will be trained in a 6 hours course on how to carry out the intervention. The training is going to be organized as interactive workshops and implemented through one to three "bed side" trainings at the ward. We refrained from administering the intervention program solely by research nurses because we want to assess our intervention in a pragmatic way and integrated in nurses' daily schedule.

Patients from the control group will receive standard care. Standard care includes standard pharmacological pain treatment, but neither standardized teaching or application of written materials, nor other evidence based treatment protocols are given.

### Outcomes

The effectiveness of SCION-PAIN program will be assessed by the following outcome measures at the individual patient level:

Primary outcome is the difference in patient related barriers to management of cancer pain (Barriers Questionnaire II) between control and intervention group [13].

The secondary outcome measures include:

1. intensity of pain (sensory dimension)

2. impact of pain and interference of pain in the patient's life (reactive dimension) [15]

3. Coping with pain [16]

4. Adherence to pain medication [17] and

5. HRQoL [18]

The outcome measures above will be assessed at trial inclusion, at discharge and 7, 14 and 28 days after discharge. The effectiveness of SCION-PAIN program will be assessed on an individual patient level by comparing the patient related barriers at day 7 after discharge in control versus intervention group.

### Sample size

Sample size was calculated based on the t-test (α = 0.05) for cluster randomized controlled studies [19] with an assumed clinically relevant difference of 0.4 points (SD 0,7) per BQ II item, an intra cluster correlation (ICC) of 0.05 and a power of 80%. These assumptions yield a sample size of 208 study participants. To allow some moderate drop out, 240 participants will be included into the study.

### Randomization

Participating wards will be pair matched before randomization according to their clinical profile. Randomization of wards will be performed (1) within these matched pairs, (2) concurrently for all wards prior to study, (3) by a reproducible SAS PROC PLAN code, and (4) by an external department (Institute for Medical Epidemiology, Biostatistics, and Informatics of the Martin-Luther-University Halle-Wittenberg). Thus, the allocation procedure on cluster and individual level will be concealed. The individual patients will be included according to their basic treatment of malignancies on the participating wards.

### Blinding

Nurses who administer the interventions and assess the outcomes might be aware of group allocation due to their participation in the training to manage the SCION-PAIN program. Patients are not informed about group assignment, but might be aware of it due to unmasking information from nurses. Therefore group allocation perception of included patients will be assessed at the end of trial 28th day after discharge.

### Implementation

**Figure 2:**
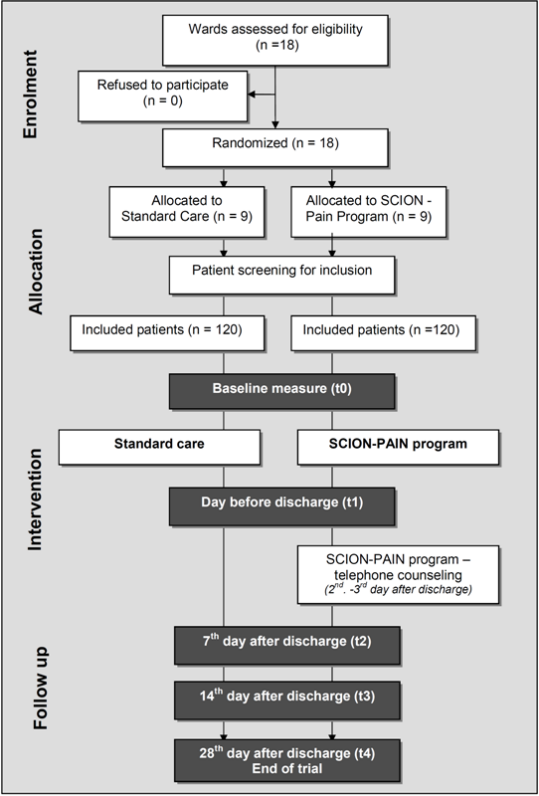
Trial profile

Adult cancer patients with persistent pain will be recruited by the nurses of the participating wards under the guidance of a research nurse. Participants will be included in the trial if they meet inclusion criteria and have signed informed consent (see Figure 2). To ensure the implementation of the intervention, quality audits will be conducted in the intervention groups in both study centers based on the guidelines of the Royal Collage of Nursing [20] and the German Network of Quality Assurance in Nursing Care. The audit will be based on (1) regular monitoring of the trained nurses' knowledge about the training module, and (2) on an additional comparison of nursing records with study documentation for 10% of all included patients.

### Statistical methods

The statistical analysis of the primary outcome patient related barriers to pain management will be conducted based on hierarchical models for the analysis of cluster randomized trials [19], including a random intercept for the respective ward. Due to our limited number of wards we a priori specified to statistically adjust the analysis by covariates (age, sex, diagnose, metastasis, performance status (ECOG), pain management/CPMI [21], pain duration, cancer treatment [22-24], depression and anxiety [7] that are significantly (P < 0.1) different at baseline. We plan to test baseline differences not routinely but because we expect significant baseline differences due to experience of previous CRT in a similar setting with 16 clusters where we found systematic and explainable differences between groups at baseline [25]. Baseline differences will be judged by simple t- and χ^2^-tests.

Subgroup analyses will be undertaken by checking interaction between well established covariates (anxiety, depression and pain intensity [7]) and primary outcome. An intention to treat approach will be used for the analysis. The trial will be reported according to the guidelines of the consolidated standards of reporting trials (CONSORT) extension for cluster randomized controlled trials [26].

### Time plan

The SCION-PAIN program is a further development of a previous self care program for management of chemotherapy induced anorexia, nausea and emesis published elsewhere [25]. The development of the special pain program began in February 2007 and was finished in June 2008. The patient recruitment started in October 2008 and will continue until December 2009. The trial will be completed in July 2010.

## Discussion

We expect SCION-PAIN program to be effective in improving patients' self management abilities of cancer pain.

Methodical strength of our study is the random group allocation minimizing known and unknown potential sources of bias.

It is hypothesized that patients who receive the multi modular structured intervention will have less patient related barriers to the management of cancer pain. Our hypothesis is based on Greens PRECEDE Model explaining health behavior [27,28]. The model was specified for pain related behavior within the conceptual framework by Yates et al. [29]. There are three categories influencing pain related health behavior identified by the model. Those are: (1) *predisposing factors*, such as beliefs, attitudes and perceptions; (2) *enabling factors*, as knowledge and skills and (3) *reinforcing factors*, i.e. feedback by family or health professionals.

The SCION-PAIN program focuses on predisposing and enabling factors because patient related barriers, such as misconceptions about opioid use, crucially interfere in establishing and maintaining a proper pain related self management [7].

**Figure 3:**
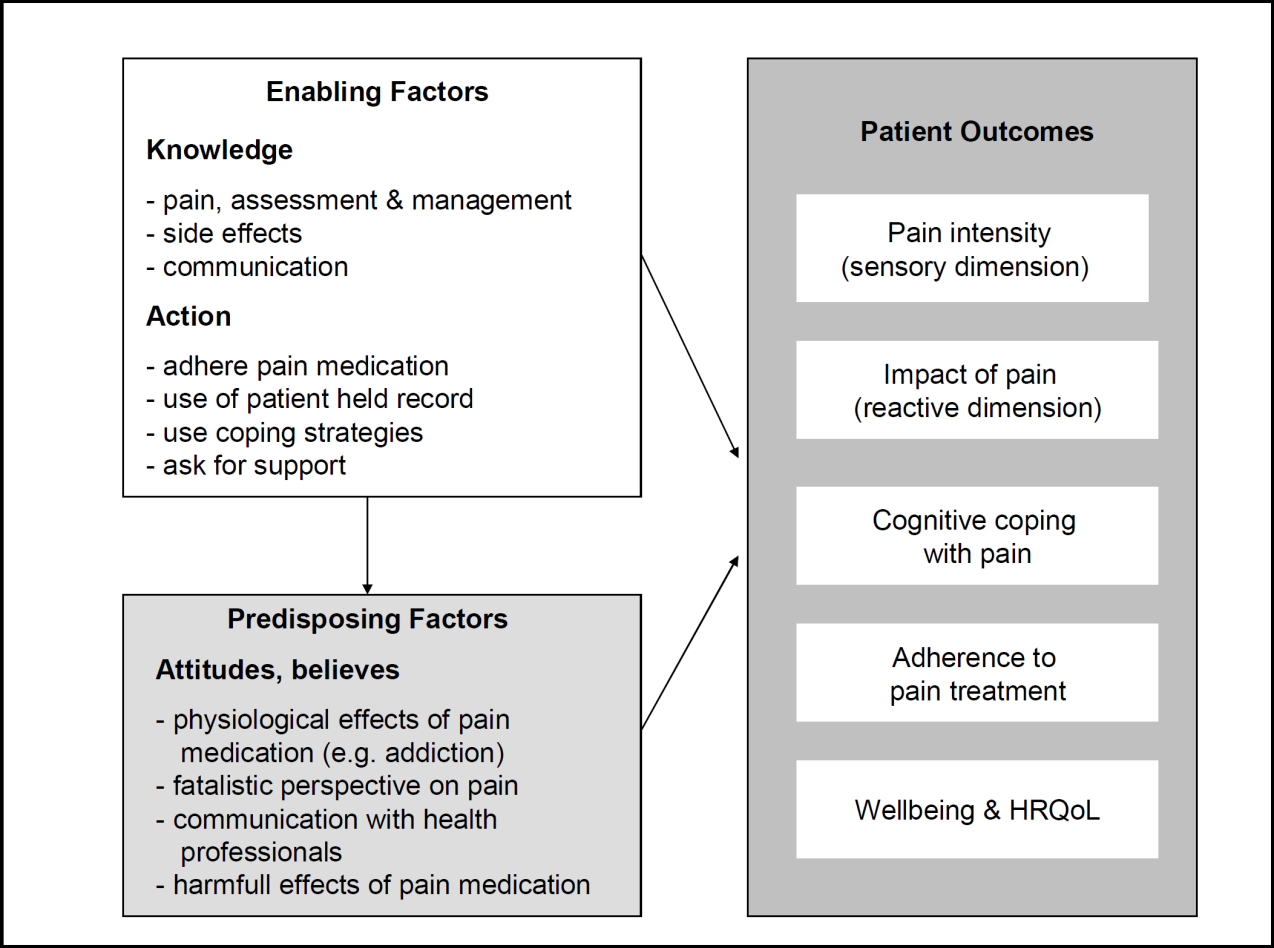
**Conceptual framework based on PRECEDE Model **[27,28]** and Yates et al**.[29].

As Figure 3 shows in variation to Green and Yates the factors we newly organized according to patients' resources for pain related self management. Therefore the SCION-PAIN program distinguishes between patients' resources regarding *knowledge, self care actions *(enabling factors) and *believes *(predisposing factors). According to this structure pain related self management will be best in patients which are *acting *(show high adherence to medication, communicate with health care professionals, and use complementary strategies) based on a sufficient body of *knowledge *and supportive positive *attitudes*.

The purpose of our trial is to contribute evidence on interventions improving self management in cancer patients with pain. As the SCION-Pain program will be integrated in nurses' daily working schedule and applied through regular ward staff, thus will be tested under "real world" circumstances, the results may be generalized to similar settings. Therefore the SCION-Pain program suggests a practical and pragmatic approach that is focused on patients' resources.

## Abbreviations

CPMI: (Cleeland pain management index); HRQoL: (Health related quality of life); PMR: (Progressive Muscle Relaxation according to E. Jacobson); PRECEDE: (Predisposing, Reinforcing and Enabling factors, and Causes in Educational Diagnosis and Evaluation); SCION: (Self Care Improvement through Oncology Nursing); ECOG: (Eastern Cooperative Oncology Group).

## Competing interests

The authors declare that they have no competing interests.

## Authors' contributions

PJ, PR, EK, OK, ATC, IH & ML were responsible for defining the research question and the drafting of the study proposal. PJ, PR, MK, ATC, IH & ML are integrated into coordination and realization of the study. PJ, MK and OK were responsible for the drafting of this paper. The final version was approved by all authors.
